# Changes in the number of suicide re-attempts in a French region since the inception of VigilanS, a regionwide program combining brief contact interventions (BCI)

**DOI:** 10.1186/s12888-020-2443-6

**Published:** 2020-01-28

**Authors:** Larissa Fossi Djembi, Guillaume Vaiva, Christophe Debien, Stéphane Duhem, Anne-Laure Demarty, Yves-Akoli Koudou, Antoine Messiah

**Affiliations:** 1 INSERM Research unit U-1178 “Mental Health and Public Health”, Centre de recherche en Epidémiologie et santé des populations (CESP), INSERM, Université Paris-Sud, Université Paris-Saclay, Hôpital Paul-Brousse, Villejuif, France; 2 SCALab Laboratory, CNRS, UMR 9193, Université de Lille, Lille, France; 3 Department of Adult Psychiatry, Hôpital Fontan, CHRU de Lille, Lille, France; 4 INSERM, Clinical Investigation Center (CIC) 1403, CHRU de Lille, Université de Lille, Lille, France; 5 UMRS 1018 Centre de recherche en Epidémiologie et santé des populations (CESP) “Epidemiology of Cancer, Genes and Environment”, Hôpital Paul-Brousse, Villejuif, France

**Keywords:** Suicide attempts, Prevention, Brief contact interventions, Evaluation

## Abstract

**Background:**

Brief Contact Interventions (BCIs) after a suicide attempt (SA) are an important element of prevention against SA and suicide. They are easier to generalize to an entire population than other forms of intervention. VigilanS generalizes to a whole French region a BCI combining resource cards, telephone calls and mailings, according to a predefined algorithm. It was implemented gradually in the Nord-Pas-de-Calais (NPC), France, between 2015 and 2018. Here, we evaluate the effectiveness of VigilanS, in terms of SA reduction, using annual data collected by participating centers. Hypothesis tested: the higher the VigilanS implementation in a center (measured by penetrance), the greater the decrease in the number of SA observed in this center.

**Methods:**

The study period was from 2014 to 2018, across all of NPC centers. We performed a series of linear regressions, each center representing a statistical unit. The outcome was the change in the number of SA, relative to the initial number, and the predictive variable was VigilanS’ penetrance: number of patients included in VigilanS over the total number of SA. Search for influential points (points beyond threshold values of 3 influence criteria) and weighted least squares estimations were performed.

**Results:**

Twenty-one centers were running VigilanS in 2018, with an average penetrance of 32%. A significant relationship was identified, showing a sharp decrease in SA as a function of penetrance (slope = − 1.13; *p* = 3*10^− 5^). The model suggested that a 25% of penetrance would yield a SA decrease of 41%.

**Conclusion:**

VigilanS has the potential to reduce SA. Subgroup analyzes are needed to further evaluate its effectiveness. Subgroup analyses remain to be done, in order to evaluate the specific variations of SA by group.

## Background

According to world health organization (WHO), more than 800,000 people die by suicide each year worldwide, corresponding to one person every 40 s [1]. No region or age group is spared. From pre-adolescence to old age, suicide concerns the whole society [2], with a devastating impact on families and loved ones that can last several years. In addition to the human consequences, suicides and suicide attempts (SA) represent an economic burden; in France, this burden amount to nearly 10 billion € per year: 88% concern the costs related to the loss of productivity caused by the victim, and 12% concern the costs related to health care and other related expenses [3]. People who have already made one or more SA present a higher risk of suicide than people who have never made a SA [1]. In 2014, 7.1% of people in France report having had at least one SA in their lifetime, of whom 36.8% had more than one SA [4]. From 2004 to 2011, there was an increase in hospital stays for SA, by 4.8% for men and 2.9% for women, and from 2010 to 2014, an increase of annual incidence SA, from 0.5 to 0.8% [4, 5].

Although there is a lot of information on the epidemiology of suicidal behavior, reducing the risk of SA is still an issue. Among the suicide prevention controlled trials, about one-third have shown and efficacy in reducing SA [6]. These trials can be divided into two approaches: first, intensive interventions, that consist in regular therapeutic sessions; and second, brief contact interventions (BCIs) after a SA, that maintain the relationship between mental health care providers and the suicide attempter. Their common goal is to help patients cope with any new suicidal crisis. BCIs have been the subject of several studies in recent years, available in two meta-analyzes. According to the results of one of these meta analyzes, BCIs were effective on the number of SA repetitions per person [7]; according to the other meta-analysis, BCIs were effective to prevent a repeat suicide attempt at 12 months [8]. Among the media used to maintain contact, there are: phone calls, focusing on patient’s mental health state and adherence to post-discharge treatment [9]; resource card delivery, with indication of number(s) to be used to call a crisis management professional if needed [10]; mailing of letters, that originate from a person who met the suicidal patient during his/her hospital stay [11]; mailing of postcards [12] and text messages (SMS), sent in order to maintain contact [13]. Several researchers have shown the efficacy of these interventions. Bertolote and coll. Found the efficacy of phone calls on suicide mortality, but did not demonstrate this effect on SA, contrasting with Cebria and coll. Who found a decrease in the number of SA reccurence related to phone calls [14, 15]. Fleischmann and colleagues found a significant reduction in death by suicide among suicide attempters, based on continuous communication in combination with standard treatments [16]. On the other hand, Moussavi and coll. Found no significant difference in reduction of SA recurrence between an intervention group receiving a phone call and a control group; there was a decrease of suicidal thoughts, however, in the intervention group [17].

Given the divergent findings between studies on SA reduction, Vaiva and colleagues proposed a composite BCI called “Algos” [6, 18]. This BCI relied on three types of contact intervention: a phone call for those who had attempted suicide several times, a series of postcards when the patient was not reachable or did not adhere to post-discharge treatment, and a crisis card for first-time attempters. A randomized controlled trial was realized on more than 1000 patients in 24 hospitals in France, comparing Algos to treatment as usual. Results from this trial led the authors and health care authorities to scale it up to the general population. Given some equivocal results from the Algos trial [18, 19], the intervention was significantly enhanced, and relabeled VigilanS (Vigilance for the prevention of Suicide recurrence).

Created in 2014 in collaboration with the Nord-Pas de Calais hospitals, and operational since 2015, VigilanS allows to recontact any suicidal person immediately after a SA, by a team of mental health care professionals specially trained in suicidal crisis management [20]. Nord-Pas de Calais (NPC) is a region marked by high rates of suicidal behavior: nationally, it ranks second for suicide deaths, with a rate of 18.1 suicides per 100,000 inhabitants [21]; it is in first place for SA (24.6 SA per 100,000 inhabitants, 54% above the national rate) [2]. From the start of the VigilanS system, a series of evaluation projects were designed, using aggregated data collected by the hospitals on the one hand, and using patients’ characteristics and follow-up data collected by the VigilanS system, on the other hand. The current article reports on a very first evaluation of VigilanS on the data collected since its implementation in NPC region.

The main objective of this study was to test the hypothesis of a correlation between the decrease of SA rate and the amount of coverage of VigilanS. Indeed, since VigilanS scales up a BCI to an entire population, a ramp-up period of few years is expected. Specifically, we studied the relationship between the SA variation and VigilanS’ penetrance (a quantity measuring the amount of VigilanS coverage, defined below) over 4 years, in the NPC Region. Our hypothesis was that the higher the VigilanS implementation in a center (measured by penetrance), the greater the decrease in the number of SA observed in this center. At a given hospital, penetrance is the proportion of people who attempted suicide and were enrolled in VigilanS, relative to all evaluated people who attempted suicide, regardless of their enrollment in VigilanS.

## Methods

The VigilanS study was authorized by the French Ministry of Health and approved by the Comité de Protection des Personnes (Ethics Committee) of Nord-Pas-de-Calais region. It was registered with ClinicalTrials.gov (NCT03134885).

### Population

The study population consists of all patients who had a SA evaluated at any hospitals in the NPC region. These hospitals were covered gradually by VigilanS system from 18 hospitals in 2015 to 21 hospitals in 2018.

Enrolment of patients in VigilanS is done by participating centers, tentatively on every patient admitted in the centers’ emergency department after a SA. Due to work load, to possible under staffing, and to patient’s declination of participation, not all eligible patients are enrolled. On a yearly basis, each center reports two numbers: 1) the number of SA included in VigilanS, and 2) the total number of SA evaluated in the center (enrolled or not in VigilanS). The ratio of these two numbers is used to calculate the penetrance (details below). When a center participates for the first year, it also transmits the number of SA it evaluated during each of the 3 previous years.

#### Enrolment into VigilanS

**Fig. 1:**
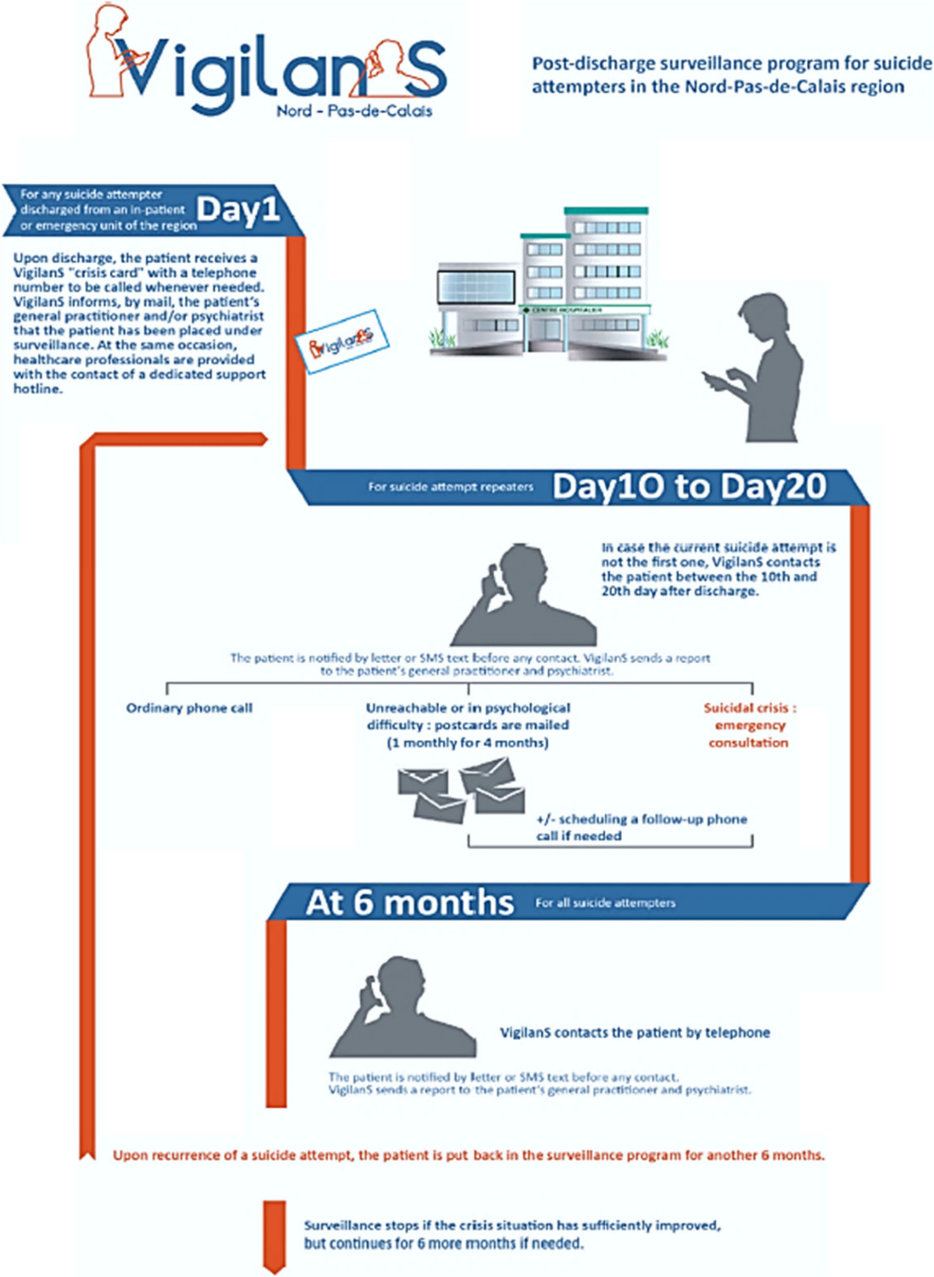
Diagram of the VigilanS system [20]

Any suicidal patient leaving the emergency room of a participating center is offered to enroll into VigilanS. An information note indicating the terms of service of VigilanS, as well as patient’s right to object, is given. If the patient accepts to participates, his/her baseline data are transmitted to a central monitoring platform [20], and (s) he receives a resource card with a single regional call number; this phone number is toll-free, either from a landline or from a mobile phone, and is available during working hours. From this point onwards, VigilanS takes charge of the intervention and patients follow-up, which complement the routine care provided by the participating centers, for a 6-month period. The description of VigilanS is summarized in Fig. 1.

#### Telephone calls between the 10th and 21st day (D10-D21)

Subjects who have done several SA are systematically called by phone between day 10 and day 21 after discharge from hospital. If the patient is not reachable, personalized postcards are sent once a month for 4 months. During the D10-D21 call, decisions are made, depending on the case at hand as judged by the calling professional: an emergency or a regular appointment is planned; a new phone call is scheduled; personalized postcards are sent; these actions can be combined; or no further action is planned.

#### Other telephone calls during follow-up

During the 6-month follow-up period, incoming or outgoing calls can also be placed, without restriction regarding their number or duration. They result in the same decision scheme as for the D10-D21 call: emergency or regular consultation, mailing of post-cards, additional follow-up call, combined actions, or no further action. Incoming calls are usually long calls from patients who need help and/or a listening ear. Outgoing calls are those planned and made by VigilanS staff.

#### 6-month calls

For all patients included, a telephone call is scheduled at the end of the 6th month post-discharge, in order to perform a clinical check-up. The VigilanS program stops at 6 months if there has been no new SA during this period. If judged necessary by the calling clinician, the program can be extended for another 3 or 6 months. In case of a new SA during the follow-up period, the entire VigilanS program is reset for another 6 months. If a patient reiterates a SA after the follow-up period, (s) he re-enters VigilanS. There is no limit on the number of entries.

### Data processing

The yearly number of SA evaluated in each center and the number of patients included in VigilanS are recorded in a database, which also contains the number of SA for the years before the start of VigilanS. For the purpose of the current study, the outcome was the SA variation from 2014 to 2018, and the explanatory variable was the penetrance of VigilanS over the 2015–2018 period, as follows.

- The relative SA variation from 2014 to 2018 is the difference between the number of SA in 2014 and in 2018, over the number of SA in 2014. The year 2014 is the year before VigilanS was created. This variable informs us about the changes in SA since the inception of Vigilans, in 2015, in each center.
  $$ SA\  Variations\ 2014\  to\ 2018=\frac{\ \mathrm{Suicides}\ \mathrm{Attempt}\mathrm{s}\ 2018-\mathrm{Suicides}\ \mathrm{Attempt}\ 2014}{\mathrm{Suicides}\ \mathrm{Attempt}\ 2014} $$
- The penetrance of VigilanS in a given center over 4 years is the total number of SA included in VigilanS from 2015 to 2018 over the total number of SA from 2015 to 2018.
  $$ Penetrance=\frac{\mathrm{SA}\ \mathrm{in}\ \mathrm{VigilanS}\ 2015+\mathrm{SA}\ \mathrm{in}\ \mathrm{VigilanS}\ 2016+\mathrm{SA}\ \mathrm{in}\ \mathrm{VigilanS}\ 2017+\mathrm{SA}\ \mathrm{in}\ \mathrm{VigilanS}\ 2018}{\mathrm{SA}\ 2015+\mathrm{SA}\ 2016+\mathrm{SA}\ 2017+\mathrm{SA}\ 2018} $$

The value of SA in VigilanS 2015 is set to zero for a center starting VigilanS in 2016, and the values of SA in VigilanS 2015 and 2016 are set to zero for a center starting VigilanS in 2017.

### Statistical analysis

We used a linear regression (Y = α * X + β) where the dependent variable Y was SA variation and the independent variable X was VigilanS penetrance. As with any linear regression modeling, we had to preform two specific sub-analyzes: first, the search for influential points, with a possible exclusion of these points; and second, the residual analysis, to determine if the model was adequate and if the homoscedasticity hypothesis was respected.

For the analysis of influential points, we used 3 criteria: “Hatvalue”, “DFBetas” and “DFFitts” [22]. A point was considered as influential if it was positive for these 3 criteria (above the respective thresholds).

#### Hatvalues

These values are common measure of leverage. They measure of how far an observation is from the others in terms of the levels of the independent variables. In simple regression, they measure the distance of point i from the mean of the predictive variable [22]. The values are between 1/n and 1, and the threshold recommended by Belsley, Kuh and Welsch is $2\ast \frac{\left(\mathrm{p}+1\right)}{\mathrm{n}}$ .

$$ {h}_i=\frac{1}{n}+\frac{\left({x}_i-\overline{x}\right)2}{\sum \left(x-\overline{x}\right)2}\kern2em i=1\dots, n;\kern2em x= explanatory\ Variable;\kern2em \overline{x}= mean\ of\ explanatory\ Variable $$

#### DFBetas

They measure the difference in each parameter estimate, with and without each point. These values ​​examine how the regression coefficients (α and β) change if the influential value is omitted from the model. DFBetas’ large values indicate observations that influence α and β estimations [22]. The threshold recommended by Belsley, Kuh and Welsch to indicate influential observations is 2/√n.

$$ DFBeta{s}_i=\frac{B-{B}_{\left(-i\right)}}{S_{\left(-i\right)}\sqrt{{\left({X}^TX\right)}^{-1}}}\kern2em {\left({X}^TX\right)}^{-1}=1/n\sum x{i}^2-\left(\sum xi\right)2 $$

- *B: regression coefficient* (α or β) *obtained with all the data;*
- *B*_(−*i*)_: *regression coefficient* (α or β) obtained when the observation « *i* » is deleted
- *S*_(−*i*)_ *Standard error estimate without the observation « i » i=1…, n;*
- *α = slope; β = intercept*
- *(X*^*T*^*X)*^*−1*^*=1/n Σxi*^2^*−(Σxi)*^2^

#### DFFitts

Proposed by Welsch and Kuh (1980), DFFitts is the difference between the predicted value obtained with the complete data and the value obtained after removing the influential observation [22]. It quantifies, in number of standard deviations, how much the predicted value changes when that observation is omitted. An observation is influential if the absolute value of its DFFITS is greater than $2\ast \frac{\sqrt{\left(\mathrm{p}+1\right)}}{\left(\mathrm{n}-\mathrm{p}-1\right)}$ .

$$ DFFitt{s}_i=\frac{\hat{y}-{\hat{y}}_{\left(-i\right)}}{S_{\left(-i\right)}\sqrt{h_i}} $$

- ŷ − ŷ_(−*i*)_: *difference of the predicted values obtained at inclusion and exclusion of the observation* “*i*”
- *S*_(−*i*)_ *Standard error estimate without the observation « i » ; h*_i_ *: hat value of observation « i » ; i=1,…, n*

Then the relationship between the SA variation and the penetrance in VigilanS was determined by linear regression with parameter estimation using the weighted least squares method (WLS). Normally, Ordinary Least Square method (OLS) is valid if the variance of errors is constant (homoscedasticity). When this hypothesis is not respected, one solution is to use WLS.

Here, the weighting was done on size of each center (SA 2018), in order to have a homogeneity (see Appendix).

To determine the gain of using WLS, we compared the models before (OLS) and after weighting (WLS), using two criteria: explanatory power and predictive power. A model has a good explanatory power if the percentage of variance explained by *R*^*2*^ (determination coefficient) is close to 1; it has a good predictive power if the relationship between the explanatory variable (X) and the outcome (Y) is significant at 5% (the slope α is significantly different from 0).

The analyses were performed using software R version 3.4.3.

## Results

**Fig. 2:**
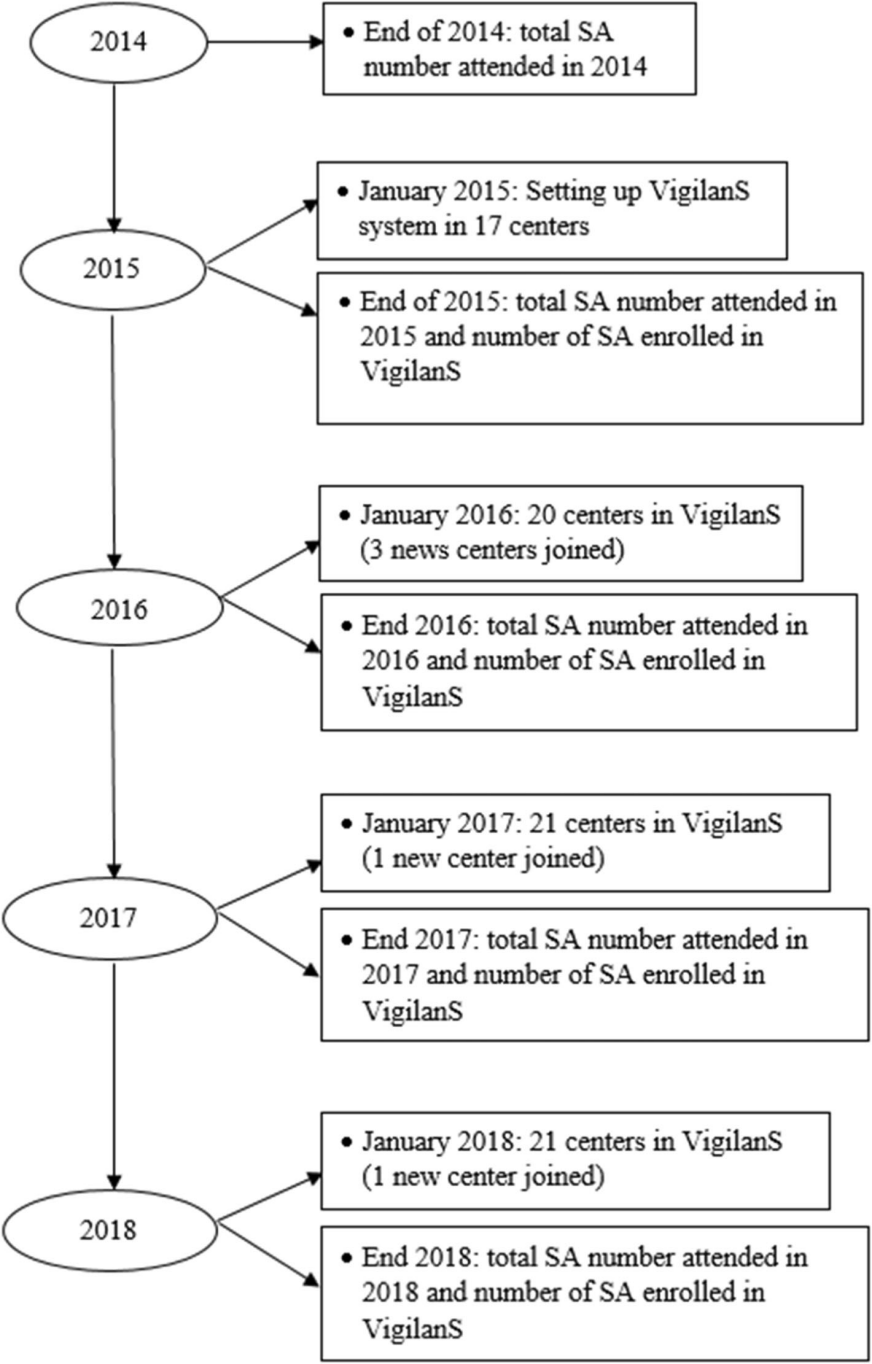
VigilanS summary during the study period 2014–2018

By 2018, 21 centers were participating in VigilanS: 17 since 2015, 3 since 2016, and 1 center since 2017 (Fig. 2).

**Fig. 3:**
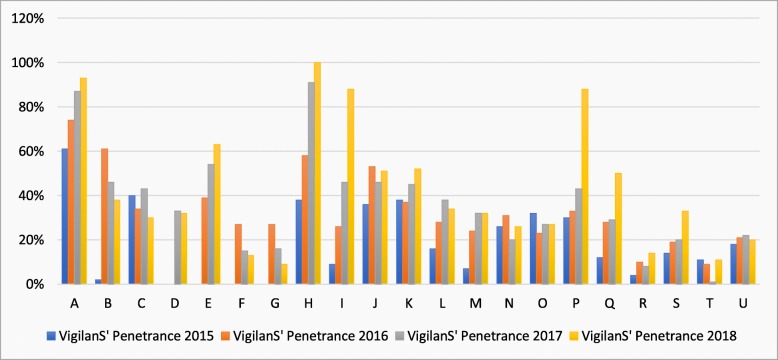
Penetrance in VigilanS in each year, by centers

At the center level, penetrance increased over the years: centers with penetrance above 0.3 were 33% in 2015, 43% in 2016, 57% in 2017, and 67% in 2018 (Fig. 3). When penetrance was computed over the 4 years, from 2015 to 2018, we found that 9 out of 21 centers achieved a penetrance of 0.3 or above.

### Search for influential points

**Fig. 4:**
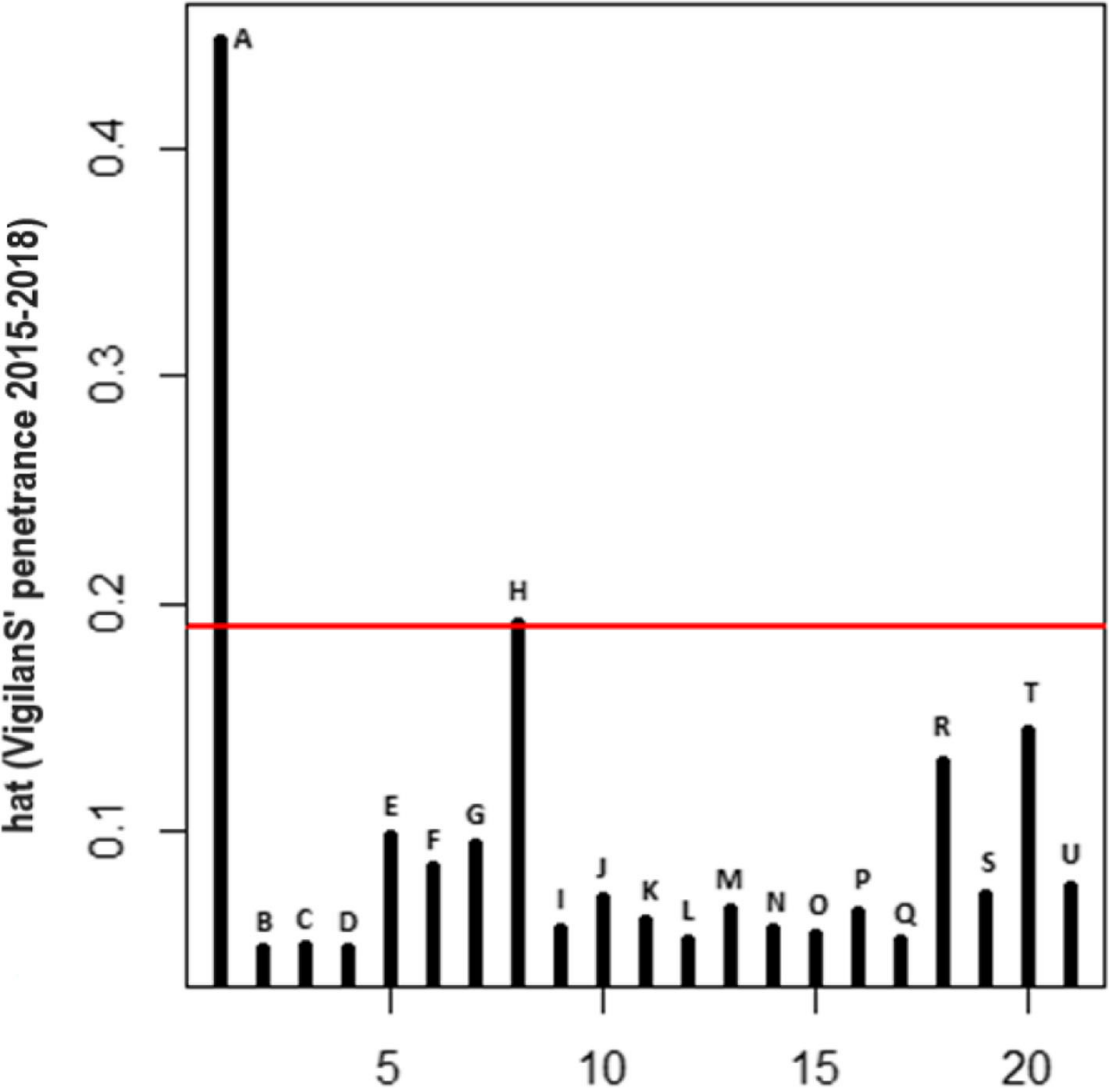
Graph of influential points 2014–2018 (Hatvalues)

**Fig. 5:**
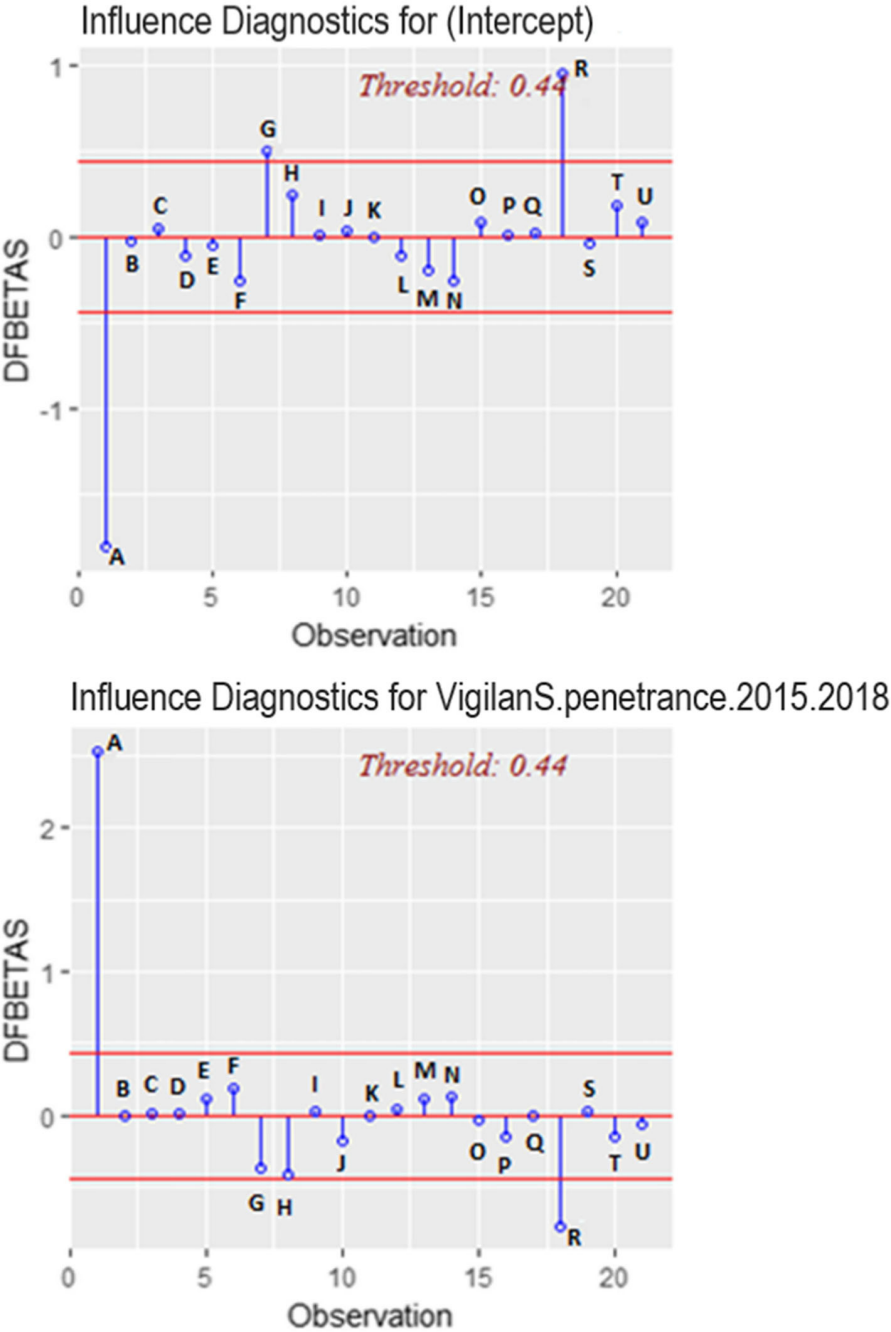
Graph of influential points, 2014–2018 (DFBETAS)

**Fig. 6:**
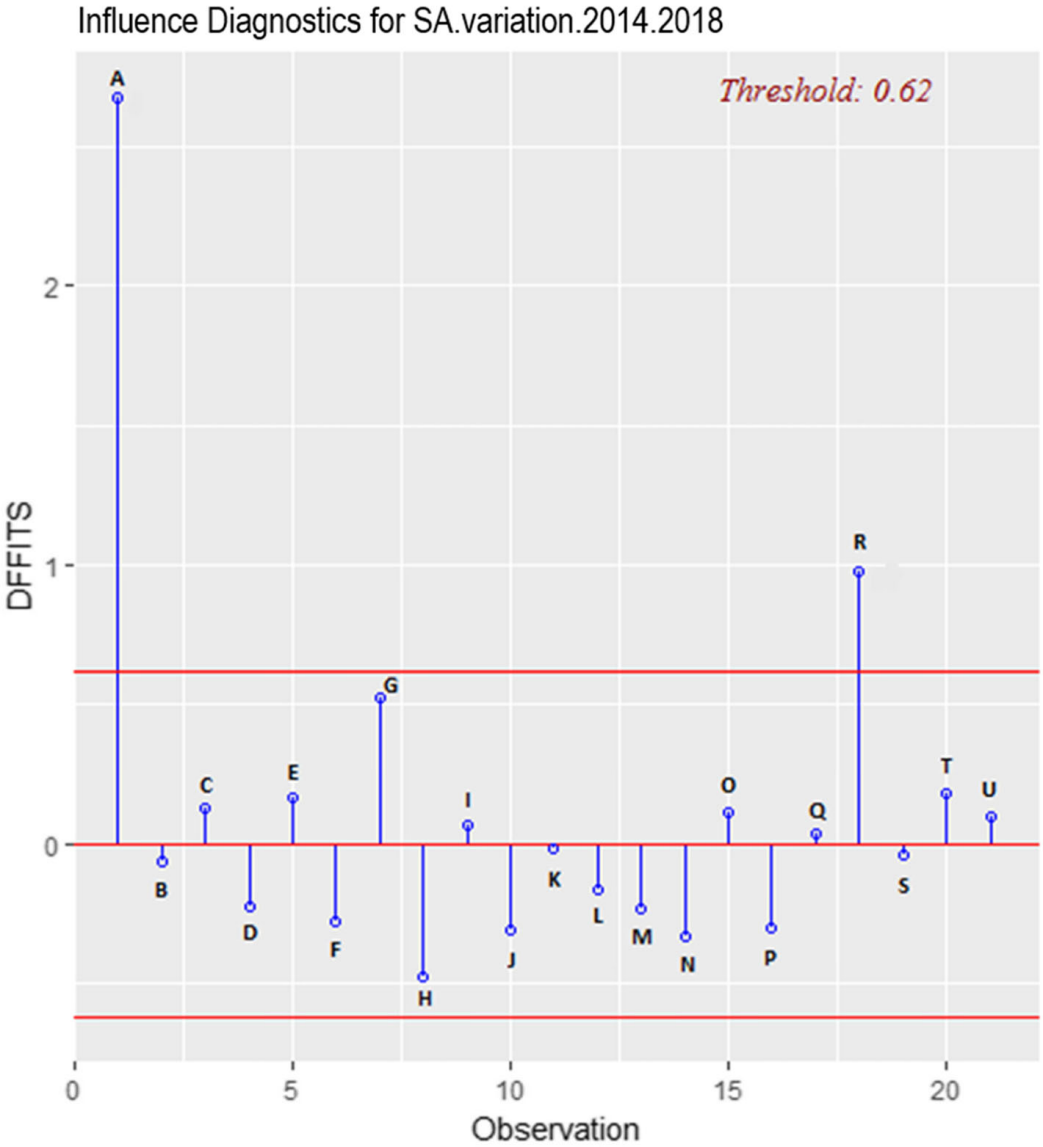
Graph of influential points, 2014–2018 (DFFITS)

On the basis of the three criteria, Hatvalues (Fig. 4), DFBetas (Fig. 5) and DFFitts (Fig. 6), the influential point was point A. This was one of the centers that had been present since the system opened in 2015, and had a higher penetrance compared to all other centers. It was subsequently excluded from the linear modeling.

### Comparison before and after removing the influential point

**Fig. 7:**
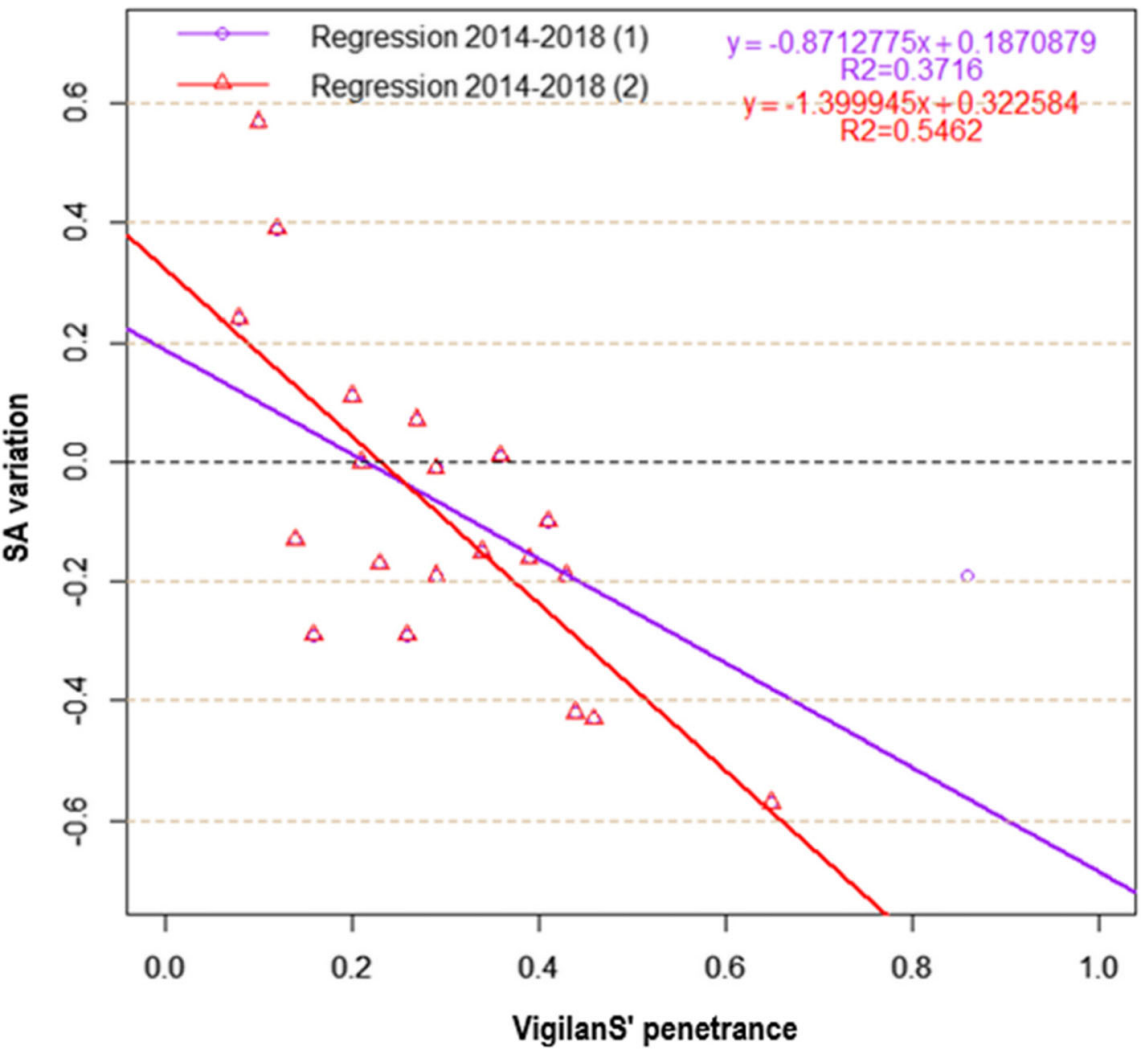
Linear regressions before and after removing the influential point, 2014–2018

From the graph and the different regression parameters (Fig. 7 and Table 1), we can see that model 2 performs better after the exclusion of the influential point. Compared to model 1, it has better explanatory power, (*R*^*2*^ = 0.54 > *R*^*2*^ = 0.37) and better predictive power (*p* = 2.10^− 4^ < *p* = 0.0033).

**Table 1:** Summary of regression estimators before and after withdrawal of the influential point, 2014–2018

	Coefficients							Residual Std. error		*R*^*2*^	Fischer
	a = penetrance			b = Intercept							
	Estimate	Std. error	T value	p	Estimate	Std. error	T value	p			
Reg 2014–2018 (1)	−0.8713	0.2599	−3.352	0.0033	0.1871	0.0956	1.957	0.0651	0.2187	0.3716	0.0033
Reg 2014–2018 (2)	−1.3999	0.3007	−4.655	0.0002	0.3226	0.0974	3.310	0.0039	0.1903	0.5462	0.0002

### Comparison of ordinary vs. weighted regressions (OLS vs. WLS)

**Fig. 8:**
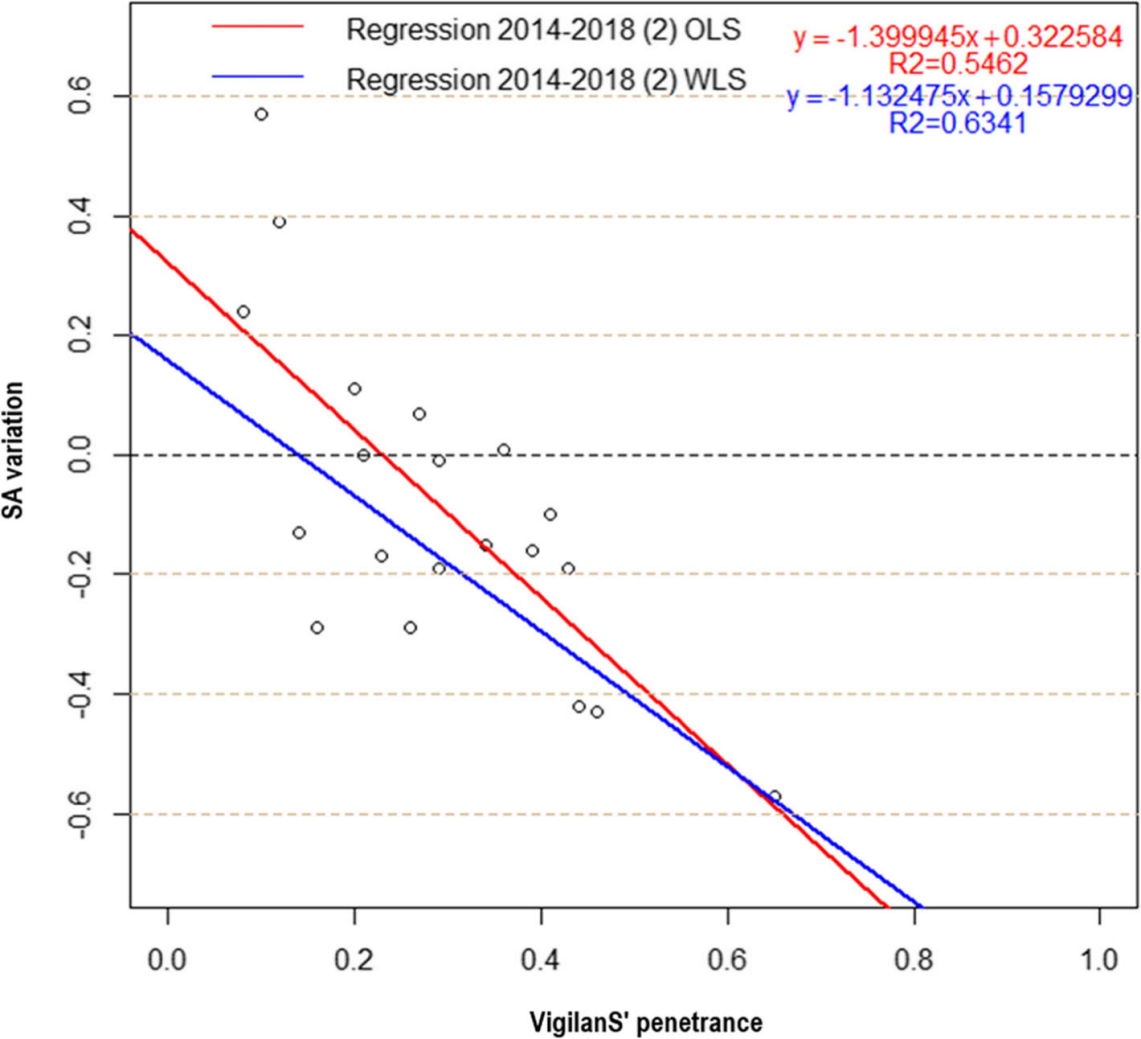
OLS and WLS regressions, 2014–2018

The WLS model was preferred over the OLS model (Fig. 8 and Table 2): it had a better explanatory power (*R*^*2*^ = 0.63 > *R*^*2*^ = 0.54), and a better predictive power (*p* = 3.10^− 5^ < *p* = 2.10^− 4^).

**Table 2:** Summary of OLS and WLS regression estimators, 2014–2018

	Coefficients								Residual Std. error	*R*^*2*^	Fischer
	a = penetrance				b = Intercept						
	Estimate	Std. error	T value	p	Estimate	Std. error	T value	p			
OLS 2014–2018 (2)	−1.3999	0.3007	−4.655	0.0002	0.3226	0.0974	3.310	0.0039	0.1903	0.5462	0.0002
WLS 2014–2018 (2)	−1.1325	0.2028	−5.585	3.10^−5^	0.1579	0.0697	2.267	0.0359	0.0006	0.6341	3.10^−5^

**Fig. 9:**
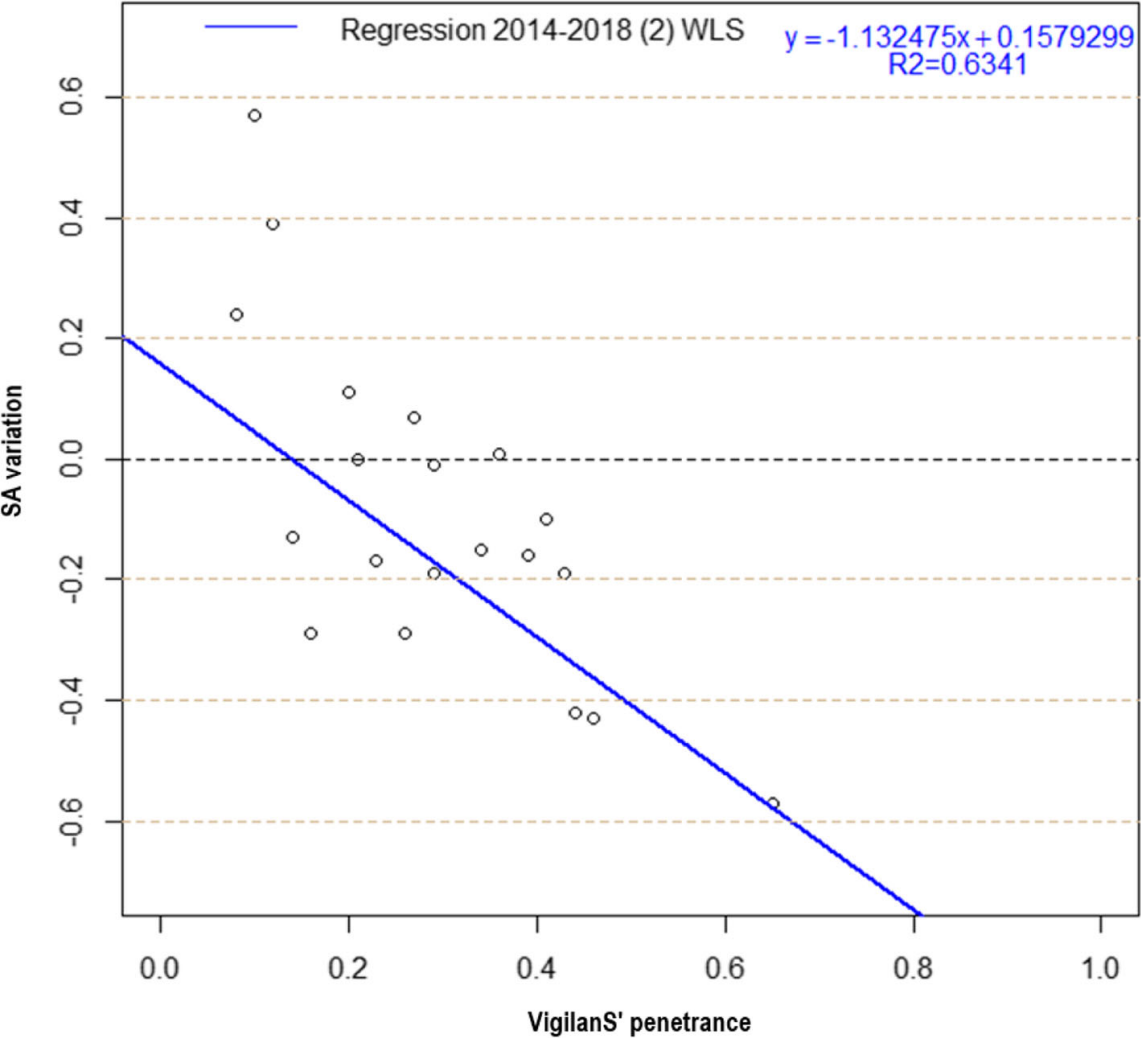
WLS regression, 2014–2018

This final model (removal of point A and WLS) showed a significant relationship between penetrance of VigilanS and SA reduction, with highest penetrance values corresponding to largest SA reduction (Fig. 9). Using the regression equation, one could derive the following:

- Including 25% of suicide patients in VigilanS results in a 12% decrease of SA.
- Including 50% of suicidal patients in VigilanS, results in a 41% decrease of SA.
- Including 75% of suicidal patients in VigilanS, results in a 69% decrease of SA.

## Discussion

The objective of this study was to examine one aspect of the effectiveness of the VigilanS system on the SA reduction, from 2014 to 2018. First, we determined the VigilanS’ penetrance each year, by center: number of SA patients enrolled in VigilanS (at the site) over the total number of SA (at that site). All the centers in NPC region included patients in the system, without being obliged to do so, a sign of shared interest on the part of the territory’s healthcare actors. In 2018, most centers had at least 20% of their suicidal patients followed by VigilanS, of which more than a third had a penetrance above 40%. This shows a good degree of VigilanS coverage on any site, although it entails enrollment work in addition to standard care. Importantly, the VigilanS team, which is experienced in crisis management, maintains contact with the patient after a SA, beyond what would occur with standard care. VigilanS was gradually implemented in all the centers in the region, from 2015 to 2017. In most centers, penetrance increased from 1 year to the next.

We performed a linear regression analysis to test the relationship between SA variation and VigilanS’ penetrance, using weighted least squares to account for the variance of residual errors. A statistically significant relationship was identified, showing a significant decrease in SA associated with higher penetrance. According to the regression, enrolling 80% of suicidal patients in VigilanS would result in a 75% decrease in SA, and in the ideal case of 100% penetrance, the decrease would be 97%. These results thus highlight the effectiveness of the VigilanS system in reducing SA. The beneficial effects of this intervention are based on follow-up of suicidal patients; the first 6 months after discharge from hospital represents a critical period of suicide and suicidal recurrence, an important period to prevent suicidal behavior. According to the study by Vuagnat and colleagues, 75% of suicidal recurrences occur within the first 6 months [23]. Maintaining contact with a suicidal patient, directly after a SA, strengthens the patient’s social bond, and gives to the patient the feeling of being seen and heard by someone [24].

Our results have similarities with those of studies that compare intervention group versus control group, where interventions are regular mail, telephone calls and long-term follow-up after discharge from emergencies [9, 14, 25–28]. In an investigation conducted for ten consecutive years, Pil and colleagues showed that phone follow-ups avoided 36% of SA and therefore reduced treatment costs [28]. According to Gruat and colleagues, the combination of the resource card and the telephone call yields a significant reduction in the number of repetitions of SA, but the telephone call is more effective among people who have already made a SA [25]. It also allows the detection of people at high risk of new SA and timely referral for emergency care within the first few weeks [9] .

By contrast, in the study by Mouaffak and colleagues, which combined the remittance of an emergency card, mailing of letters, telephone calls and regular medical treatment, there was no significant difference in the reduction of SA between the intervention and control groups. According this study, calling the patient quickly after a SA is not enough, frequent contacts must also be maintained during the first weeks post-SA in order to obtain a favorable result [29].

### Limits and positive points

Our results might be overly optimistic. They were bases on the first 4 years of implementation, that might correspond to the VigilanS’ period of maximal effect, while the patients who are the most susceptible to vigilanS’ effect are in large number. VigilanS’ effect might wear off over time, as that population of susceptible patients who still attempt suicide decreases. In addition, the final model scatter plot suggests a curvilinear relationship, challenging the linear model approach used for a possibly nonlinear relationship. The advantage of our approach is the ease of its realization, doable for a clinician with little training in statistics, as opposed to more complex modeling.

Furthermore, all centers had not been present since VigilanS’ inception in 2015. Three centers were included after 2015: 2 centers in 2016 and 1 center in 2017. The calculation of VigilanS’ penetrance over 4 years for these 3 centers only takes into account SA included in VigilanS over 2 or 3 years, Centers participating in VigilanS from the beginning might not yield the same results as those participating later, at equivalent penetrance level. In addition, our approach used the center as the statistical unit of analysis; it didn’t consider the patients’ characteristics. We don’t know how the relationship between penetrance of VigilanS and SA reduction might vary with these characteristics. We were not able to adjust for patient-level confounders, and to control the differences across centers with regards to staffing characteristics, access to mental health resources, and other possible confounders, which could influence the reduction in SA. Further analyses are needed to address this limitation.

Despite these limitations, our results suggest that the VigilanS system is promising for reducing SA. The ease of implementation of our method, the quickness of data availability, management and analysis - which is faster than in an individual, patient-based approach –, are all positive aspects for a first analysis. In addition, unlike experimental studies, we worked on real life data, on a full upscale BCI.

The aim of this article was to present a real life, population-scaled intervention and evaluate its overall effect, using a method simple enough to be easily performed by clinicians with little statistical training. However, further analyses are needed to go beyond the study limits. Subgroup analyses remain to be done, in order to evaluate the specific variations of SA by group.

## Conclusion

VigilanS is a monitoring system combining several types of BCIs, aiming at maintaining the relationship of suicidal patients with healthcare providers, during a pre-defined period, as soon as the patient leaves the hospital. The aim of this study was to evaluate its overall effect in terms of SA variation. According to the results of our analysis, VigilanS can be an effective system for SA reduction. In view of the limitations of our study, additional evaluations must be carried out in order to assess the possible effectiveness of VigilanS.

### Supplementary information

**Additional file 1.** Dataset containing all variables used to produce results of this article.

## Figures and Tables

**Fig. 10:**
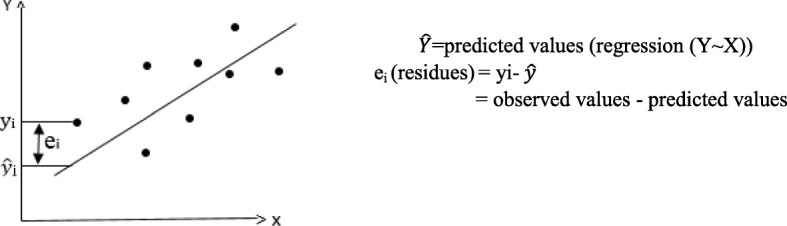
Example of linear regression of variable Y on variable X

**Fig. 11:**
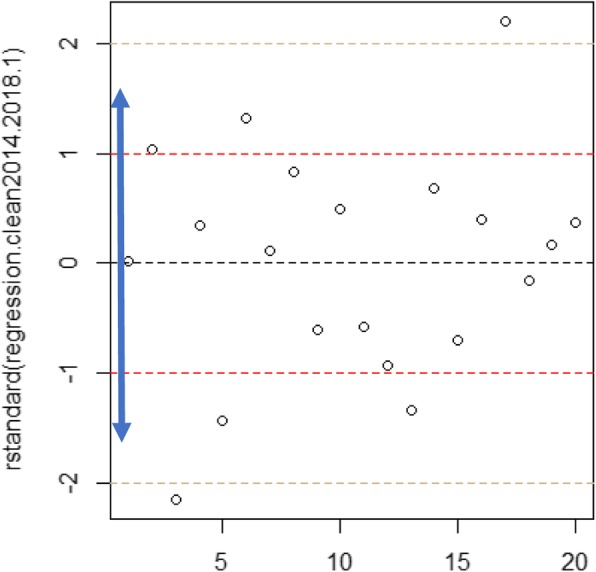
Residues standard chart before weighting (OLS), 2014-2018

**Fig. 12:**
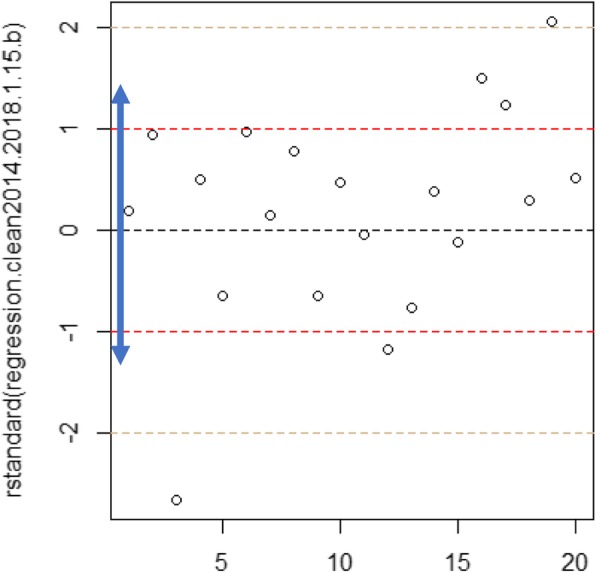
Residues standard chart after weighting (WLS), 2014-2018

## Data Availability

All relevant data are within the paper and its supplementary information file (Additional file 1).

## Appendix

### Explanation of the least squares formula used in the analysis

Let X be the predictive variable and Y the variable to be Predicted

The model (y|x) is the regression (Y~X), with the equation Y=αX+β

e_i_^2^ is a variance estimate and |e_i_| is a standard deviation estimate (σ_i_)

The ordinary least squares method assumes that there is a constant variance of errors, a homogeneous dispersion (homoscedasticity). The weighted least squares method is used when the assumption of constant variance of errors is violated (heteroscedasticity). Residual analysis aims at verifying whether this variance is constant. If the residuals don’t have a constant variance, the weighted least squares method is applied.

There are circumstances where the weights are known:

- If the *i*-th response is an average of *n*_*i*_ observations, then Var(*y*_*i*_) = σ2/ni and *w*_*i*_ = *n*_*i*_.
- If the *i*-th response is a total of *n*_*i*_ observations, then Var(*y*_*i*_) = niσ2 and *w*_*i*_ =1/ *n*_*i*_.
- If variance is proportional to some predictor *x*_*i*_, then Var(*y*_*i*_) = xiσ2 and *w*_*i*_ =1/ *x*_*i*_.

In other circumstances, the weight is determined by the shape of the residual plot.

### Graphs of the residues of our study

After weighting, the residual variance is lower than before (0.00062<0.1903, see Table 2). This show that, on average, the regression line is well adapted to the data, the hypothesis constant variation is acceptable.

## Abbreviations

BCI: Brief contact interventions
NPC: Nord-Pas-de-Calais
OLS: Ordinary Least Square
SA: Suicide attempt
WHO: World Health Organization
WLS: Weighted least squares
